# *Dnmts* and *Tet* target memory-associated genes after appetitive olfactory training in honey bees

**DOI:** 10.1038/srep16223

**Published:** 2015-11-04

**Authors:** Stephanie D. Biergans, C. Giovanni Galizia, Judith Reinhard, Charles Claudianos

**Affiliations:** 1 Queensland Brain Institute, The University of Queensland, Australia; 2 Neurobiologie, Universität Konstanz, Germany; 3 Monash Institute of Cognitive and Clinical Neuroscience, Faculty of Biomedical and Psychological Sciences, Monash University, Australia

## Abstract

DNA methylation and demethylation are epigenetic mechanisms involved in memory formation. In honey bees DNA methyltransferase (Dnmt) function is necessary for long-term memory to be stimulus specific (i.e. to reduce generalization). So far, however, it remains elusive which genes are targeted and what the time-course of DNA methylation is during memory formation. Here, we analyse how DNA methylation affects memory retention, gene expression, and differential methylation in stimulus-specific olfactory long-term memory formation. Out of 30 memory-associated genes investigated here, 9 were upregulated following Dnmt inhibition in trained bees. These included *Dnmt3* suggesting a negative feedback loop for DNA methylation. Within these genes also the DNA methylation pattern changed during the first 24 hours after training. Interestingly, this was accompanied by sequential activation of the DNA methylation machinery (i.e. Dnmts and Tet). In sum, memory formation involves a temporally complex epigenetic regulation of memory-associated genes that facilitates stimulus specific long-term memory in the honey bee.

DNA methylation is an epigenetic mechanism involved in regulating transcription in various processes from development to behavioural plasticity. DNA methylation describes the addition of a methyl-group to the 5’ position of cytosines catalyzed by DNA methyltransferases (Dnmts) forming methylcytosine. Three different Dnmts have been described: Dnmt1 (maintenance Dnmt) has a preference to methylate hemimethylated DNA; Dnmt3 (*de novo* Dnmt) methylates unmethylated DNA^1^,^2^; and Dnmt2 methylates tRNA but not DNA^3^. Besides methylation, a mechanism for active *de*methylation exists mediated by enzymes of the Tet (ten-eleven translocation methylcytosine dioxygenase) family. Tet enzymes oxidize methylcytosine to hydroxymethylcytosine which is further converted to unmethylated cytosine^4^.

DNA methylation is crucial for memory formation, as demonstrated in a number of organisms (e.g. honey bees, mollusks and rodents), and learning paradigms^5^. Tet-mediated DNA demethylation is involved in the regulation of long-term memory formation as well^6^,^7^.

Memory formation in the honey bee, *Apis mellifera*, has been well characterized behaviorally, physiologically and molecularly^8^,^9^, but regulatory processes are little understood. The proboscis extension response (PER) is commonly used in appetitive classical conditioning, with odours as conditioned stimuli (CS) and sugar water as reward (unconditioned stimulus, US)^10^. Following training honey bees form a transcription and translation dependent long-term memory^11^. Memory-associated gene expression changes have been shown in genome wide transcription studies, and consistently show downregulation of genes associated with memory formation 24 hours and longer after training^12^,^13^,^14^. Among the genes reported to be involved in memory formation are key synaptic and structural genes such as *neurexinI* and *actin*^14^,^15^,^16^.

Honey bees have three *Dnmt* genes that are also found in vertebrates: two copies of *Dnmt1,* one copy of *Dnmt2*, and one copy of *Dnmt3*^17^,^18^. Honey bees also have a functional homolog of the demethylation gene *Tet* found in vertebrates^19^ and there is direct evidence for hydroxymethylation in bees^19^,^20^. The presence of DNA methylation and DNA hydroxymethylation as well as that of the full DNA methylation machinery indicates the demand of tight regulation of gene expression in honey bees.

In honey bees DNA methylation is crucial during caste and subcaste development^21^,^22^,^23^,^24^,^25^,^26^,^27^. DNA methylation also impairs memory extinction^28^ and stimulus-specific olfactory long-term memory formation^29^. Stimulus-specific memory describes the ability of bees to form a memory that is specific to a given stimulus with a narrow generalization to other stimuli (e.g other odours). This ability is quantified as discriminatory power, which is reduced after Dnmt inhibition^29^. So far, studies looking at the effect of Dnmt inhibition in bees have used the Dnmt inhibitor Zebularine^28^,^29^. Zebularine is a cytosine mimic, which requires incorporation into DNA or RNA^30^,^31^. Another effective inhibitor, RG108, does not require incorporation into DNA or RNA^32^ and has not yet been tested in bees. Both inhibitors impair the ability of Dnmts to methylate DNA, without affecting protein or mRNA concentrations.

Here we investigated the role of DNA methylation in stimulus-specific associative long-term memory formation of bees. We show that two functionally different Dnmt inhibitors (i.e. RG108 and Zebularine) both impair stimulus-specific long-term memory formation and cause upregulation of memory-associated target genes. We investigated the temporal dynamics of *Dnmt* and *Tet* expression during the first 5 hours and at 24 hours after training, and found that *Dnmt1b*, *Dnmt3* and *Tet* are upregulated in temporally distinct patterns. Finally we show site specific methylation changes occur in several key memory-associated genes 24 hours after training.

## Results

### Dnmt inhibition impairs stimulus-specific memory formation and causes upregulation of memory-associated genes

**Figure 1:**
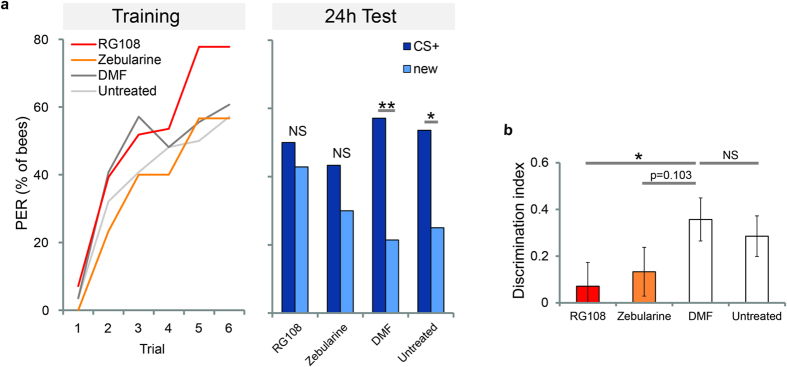
Dnmt inhibition impairs stimulus-specific memory. (**a**) The percentage of bees responding to the odorant during the training and test is shown. Bees were trained with 6 trials of odour sugar pairings and treated 2 hours after the training with Dnmt inhibitors (RG108 or Zebularine), the solvent DMF or were left untreated. The training performance of the different treatment groups was not significantly different, but there was a significant effect of training trials (glm, factor training trial p < 0.001). 24 hours after the training CS + retention and the generalisation towards a new odour were assessed. Control bees (DMF and untreated) responded significantly more to the CS + than the new odorant during the test, showing stimulus-specific long-term memory (n = 28 in both groups; McNemar test p < 0.05). This ability was impaired in bees treated with Dnmt inhibitors (n = 28 (RG108), n = 30 (Zebularine); McNemar test p > 0.05). (**b**) From the responses to the CS + and the new odorant during the test the discrimination index was calculated for each individual. Bees treated with Dnmt inhibitors had an impaired discriminatory power compared to solvent treated control bees (glm compared to DMF: RG108 p = 0.042; Zebularine p = 0.102). All data is presented as the mean (+/– SEM). ( = p < 0.1; * = p < 0.05; ** = p < 0.01; *** = p < 0.001).

We used classical absolute olfactory conditioning to study the role of DNA methylation in honey bee memory formation. Bees were trained with one odour (CS) presented 6 times followed by sugar reward. Bees were divided in four groups: one treated with the Dnmt inhibitor Zebularine, one treated with the Dnmt inhibitor RG108, one treated with the solvent (dimethylformamid, DMF), and one untreated control. Bees were treated 2 hours after training; acquisition therefore was not affected by treatment and not statistically different between groups (Fig. 1a, Training: generalized linear model (glm), factor training trial p < 0.001; factor treatment compared to DMF: RG108 p = 0.427, Zebularine p = 0.142, untreated p = 0.526; interactions trial-treatment p > 0.1). It has been shown previously that Dnmt inhibition does not affect CS + acquisition or short-term memory formation^28^,^29^, therefore we here focused on its effect on long-term memory formation. We tested for long-term memory retention 24 hours after training by presenting the trained odour (CS+) to the bees. All groups showed robust long-term memory (Fig. 1a, 24 h Test). However, associative learning also influences how individuals generalize the established memory across similar stimuli^33^. To quantify generalization we presented a novel odour (new) during the test, and recorded the response towards it. Control bees (untreated or treated with DMF) showed stimulus specific learning: they generalized weakly to the novel odour (Untreated: p = 0.029, DMF: p = 0.006, McNemar test) (Fig. 1a: 24 h Test). However, bees treated with RG108 or Zebularine showed no significant difference in the responses to the CS and novel odour (RG108: p = 0.60, Zebularine: p = 0.29, McNemar test), indicating a strong tendency to generalize across odours. We quantified the capacity to not generalize across odours using a discrimination index (Fig. 1b). RG108 treatment significantly reduced the long-term discrimination ability (p = 0.042, glm with factor treatment compared to DMF). Zebularine treatment reduced the discrimination ability to a lesser degree, close to but not reaching statistical significance (p = 0.102, glm with factor treatment compared to DMF). It however was significant in a previous study reporting the effect of Zebularine on generalization 24 and 72 hours after olfactory conditioning^29^. The inhibitor RG108 has a different molecular mechanism of inhibiting Dnmts, but had the same behavioral effect as Zebularine: good learning, but increased generalization. These results corroborate the assumption that DNA methylation is necessary for odour discrimination after olfactory learning^29^, and argue against an unknown side-effect of the drugs used.

**Figure 2:**
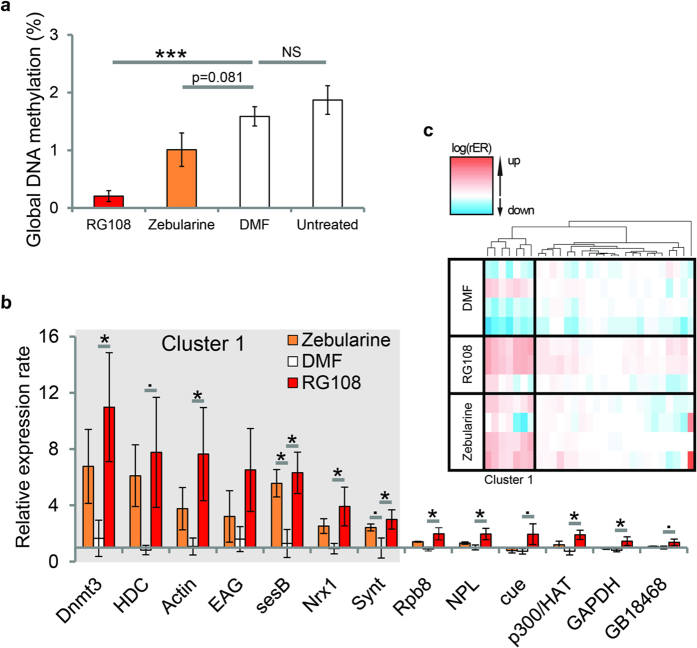
Memory-associated genes are upregulated after Dnmt inhibition. (**a**) Topical treatment with Dnmt inhibitors reduced global DNA methylation successfully in the brain confirming the effectiveness of the treatment method. RG108 was more efficient in reducing DNA methylation (n = 8 (DMF, Zebularine), n = 6 (RG108), n = 9 (Untreated); glm RG108 vs DMF p < 0.001; Zebularine vs DMF p = 0.081). (**b**) Average expression of genes sensitive to Dnmt inhibition is shown. The relative expression rate (rER) was calculated by normalising to the housekeeping gene *RPL32* and to untreated controls. Dnmt inhibition was associated with target gene upregulation. In RG108 treated bees nine genes were significantly upregulated and in Zebularine treated bees one (n = 4 (DMF, Zebularine), n = 3 (RG108, Untreated); glm factor treatment p < 0.05). The shaded box indicates genes that responded particularly strong to Dnmt inhibition, and corresponds to cluster 1 in C. (**c**) Here the rER for each gene and replicate is presented as heatmap after normalisation. Two large clusters are apparent with a cluster of 7 genes (Cluster1) being affected strongest by Dnmt inhibition. All data is present as the mean (+/– SEM). ( = p < 0.1; * = p < 0.05; ** = p < 0.01; *** = p < 0.001).

Overall RG108 and Zebularine showed similar effects on stimulus-specific long-term memory retention (Fig. 1a,b). To confirm that both Dnmt inhibitors reliably reduce DNA methylation in the brain we measured global DNA methylation 24 hours after training (Fig. 2a). We found that RG108 treatment significantly reduced global DNA methylation (Fig. 2a, p < 0.001, glm with factor treatment compared to DMF). Zebularine treatment also reduced global DNA methylation. This reduction, however, was not significant (Fig. 2a, p = 0.081, glm with factor treatment compared to DMF), confirming that Zebularine had a weaker effect compared to RG108.

Since DNA methylation regulates gene expression, we wondered which genes were most affected by Dnmt inhibition. These genes would likely to be recruited during stimulus-specific memory formation. Specifically, we quantified the expression of 30 genes that have previously been associated either with memory formation (e.g. *cAMP response element-binding protein* (*CREB), actin* and *synaptotagmin1*^14^,^15^), DNA methylation (e.g. *Dnmts*) or cell maintenance (e.g. *GAPDH*) (full gene list in: Table S1). Gene expression was measured in the brains of bees 24 hours after training for the four different treatment groups: Zebularine, RG108 or DMF treated and untreated (Fig. 2b,c) (same individuals as those presented in Fig. 1). Treatment with both RG108 and Zebularine caused a similar pattern of gene expression changes (Fig. 2b,c; glm with factor treatment and group). RG108 treatment significantly upregulated 9 genes (i.e. *Dnmt3, actin, sesB, neurexin1, synaptotagmin1, Rpb8, Npl, p300/HAT and Gapdh*) out of 30 (compared to DMF: p < 0.05, glm with factor treatment); a further 3 genes were upregulated but not significantly (i.e. *headcase, cue and GB18468*) (compared to DMF: p < 0.1, glm with factor treatment). Again, treatment with Zebularine was less effective: while generally the same genes showed a tendency for upregulation, the effect was only statistically significant for *sesB* (compared to DMF: p = 0.022, glm with factor treatment). Moreover, cluster analysis identified a group of 7 genes (i.e. *Dnmt3, headcase, actin, Eag, sesB, neurexin1 and synaptotagmin1*) that had a similar expression pattern in response to either Dnmt inhibition or solvent treatment (Fig. 2c, Cluster 1; agglomerative hierarchical clustering using Euclidian distances). These genes were notably most affected (i.e. largest expression change relative to untreated bees) by Dnmt inhibition with both inhibitors. It logically follows that these 7 genes represent good candidates for being directly targeted by Dnmts during learning. Importantly, all differentially expressed genes were upregulated in response to Dnmt inhibition. This suggests that there is a negative association between DNA methylation and gene expression in memory-associated genes (i.e. memory induces a downregulation of these genes mediated by increased methylation). Taken together, this data suggests that Dnmts regulate gene expression in a subset of memory-associated genes, acting on odour generalization rather than associative strength.

### Memory formation influences the methylation pattern of memory-associated genes 24 hours after training

**Figure 3:**
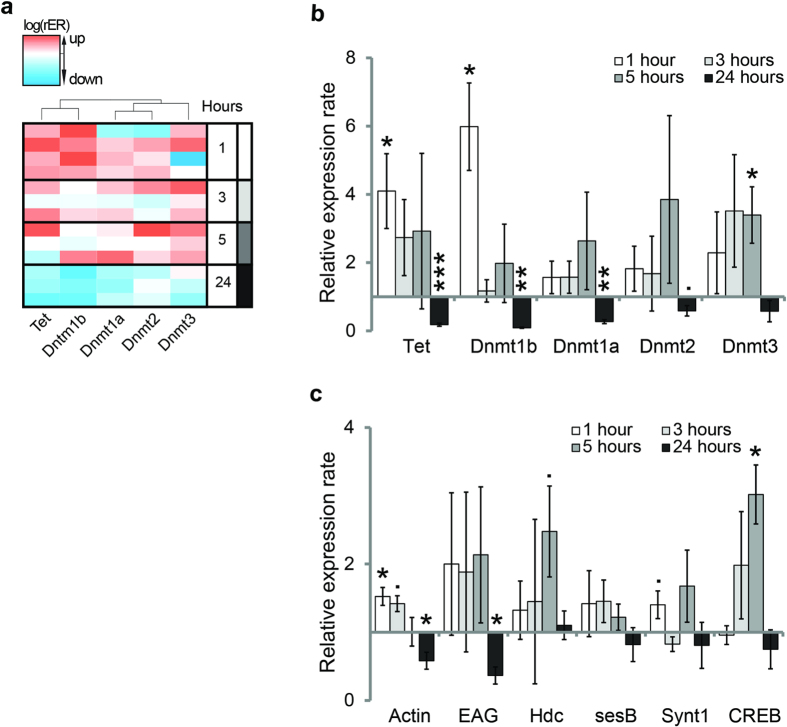
Long-term memory formation associates with distinct methylation pattern. (**a**) Overview over methylation changes and their location in target genes. Exons are displayed as black lined boxes with numbers indicating their identity (e.g. “1” – first exon in gene). Introns are indicated by black dashed lines. *Neurexin1* was too long to be fully displayed and vertical black lines in introns therefore indicate breaks. Pink boxes indicate significant changes over neighbouring CpGs and pink lines in individual CpGs, whereas solid lines indicate p-values < 0.05 and dashed lines p-value < 0.1. A differential methylation event was counted as being due to learning if the learner group was different from the unpaired or the naïve group. If the unpaired group was different from the naïve group it was counted as being due to the stimuli. Brown underlining indicates the analysed regions. (**b**) Examples for differential methylation events: The percentage of methylated cytosines at a particular genomic location is shown. Both learning and stimulation induced changes across promoters (i.e. region immediately upstream of the first exon), exons and introns (glm, factors age, season and group: * = p < 0.05, ** = p < 0.01; complete list of p-values: Table S3). Both increases and decreases in methylation were observed. The mean (+/− SEM) is presented here. (**c**) CpGs were pooled and correlation of methylation patterns between groups was calculated. The correlation coefficient for each comparison is shown here. Unpaired and naïve controls correlated more with each other than with the learning group (Spearman coefficient). (**d**) Clustering was performed by calculating Euclidian distances as input for agglomerative hierarchical clustering using Ward’s method over all CpGs. The length of tree branches indicates the distance between neighboring groups. All replicates of the learning group clustered together and separately from the two control groups, which did not form separate clusters themselves. Therefore here learning associates with a methylation pattern, which is distinct from the methylation pattern observed in untrained bees. N = 6 for all groups. (Amplicon information and bisulfite conversion efficiency: Figure S1 and S2).

We could show that Dnmt inhibition impairs stimulus-specific memory and increases the expression of a subset of memory-associated genes, suggesting that there are DNA methylation changes in the genome after learning. However, this begs the question as to where in the genome these changes occur, and how persistent they are. Therefore, we analysed DNA methylation in 11 target amplicons in 5 memory-associated genes 24 hours after training using bisulfite treatment and Sequenome mass spectrometry (Fig. 3). Changes in DNA methylation 24 hours after training would indicate that learning initiates stable changes in DNA methylation. Such changes may help to coordinate a pattern of gene expression that is needed to maintain the sensory acuity and neuronal conformation of an olfactory memory trace^34^. We analysed 4 memory-associated genes which were affected by Dnmt inhibition (*Dnmt3*, *synaptotagmin1*, *neurexinI* and *headcase (Hdc)*; Fig. 2b,c). In addition, we included *CREB,* a transcription factor frequently associated with memory formation which was not affected by Dnmt inhibition in our study. Those genes were also chosen because they were suitable for analysis with the Sequenome technology. This technology requires the design of primers with specific properties regarding melting temperature, cytosine content and size of the resulting amplicon, restricting the set of suitable genomic regions. We found that learning led to differentially methylated cytosines in all genes analysed: *neurexinI* had increased DNA methylation in exons, *Dnmt3* and *CREB* in the promoter and *Hdc* and *synaptotagmin1* both in the promoter and in one exon (Fig. 3a,b). DNA methylation not only changed in the learner group though: the “stimulated” group (i.e. CS and US presence, without temporal overlap (unpaired)) also had differentially methylated cytosines in promoters, exons and introns (Fig. 3a,b). In particular, DNA methylation increased in the *CREB* promoter region with both behavioral treatments (Fig. 3b, lower panel), confirming our previous results that Dnmt inhibition does not affect *CREB* specifically after learning.

Methylation changed bidirectionally after learning (i.e. increase and decrease) in promoters and exons (Fig. 3b upper panel). We did, however, not find evidence for DNA methylation changes in introns after learning. This could be due to the small amount of genomic regions investigated here and not necessarily reflects the true distribution of DNA methylation changes across genomic regions after learning. DNA methylation also increased and decreased after stimulation only, and unlike the learner group stimulated-only bees showed changes also in introns in addition to promoters and exons (Fig. 3b lower panel).

In order to assess the relative importance of memory formation and stimulation in driving differential DNA methylation, all investigated cytosines were pooled and analysed using a correlation and a clustering approach (Fig. 3c,d). Specifically, we treated each cytosine of the 11 amplicons analysed here as an individual data point. The group of bees that had been conditioned was less correlated with the control groups (unpaired and naïve) than those with each other (Fig. 3c, Spearman correlation). Furthermore, all six biological replicates of the learner group formed a cluster separate from the unpaired and naïve control groups (Fig. 3d, hierarchical agglomerative clustering of Euclidian distances). This indicates that memory formation influenced the DNA methylation pattern of those regions investigated here in a consistent manner, and more than simple stimulation (Fig. 3d). Although we found that stimulation alone also induces DNA methylation changes in individual CpGs, these changes were less consistent as unpaired and naïve replicates formed an intermingled cluster (Fig. 3d). This suggests a wider regulatory role of DNA methylation in brain circuitry, not limited to memory formation. Summing up, we show that memory formation (but not only) induced DNA methylation changes in some memory associated genes that persisted 24 hours after training. This could contribute to stable gene expression changes of those genes - a requisite for a long-term memory substrate.

### Dnmts and Tet are upregulated during memory formation at different time points

**Figure 4:**
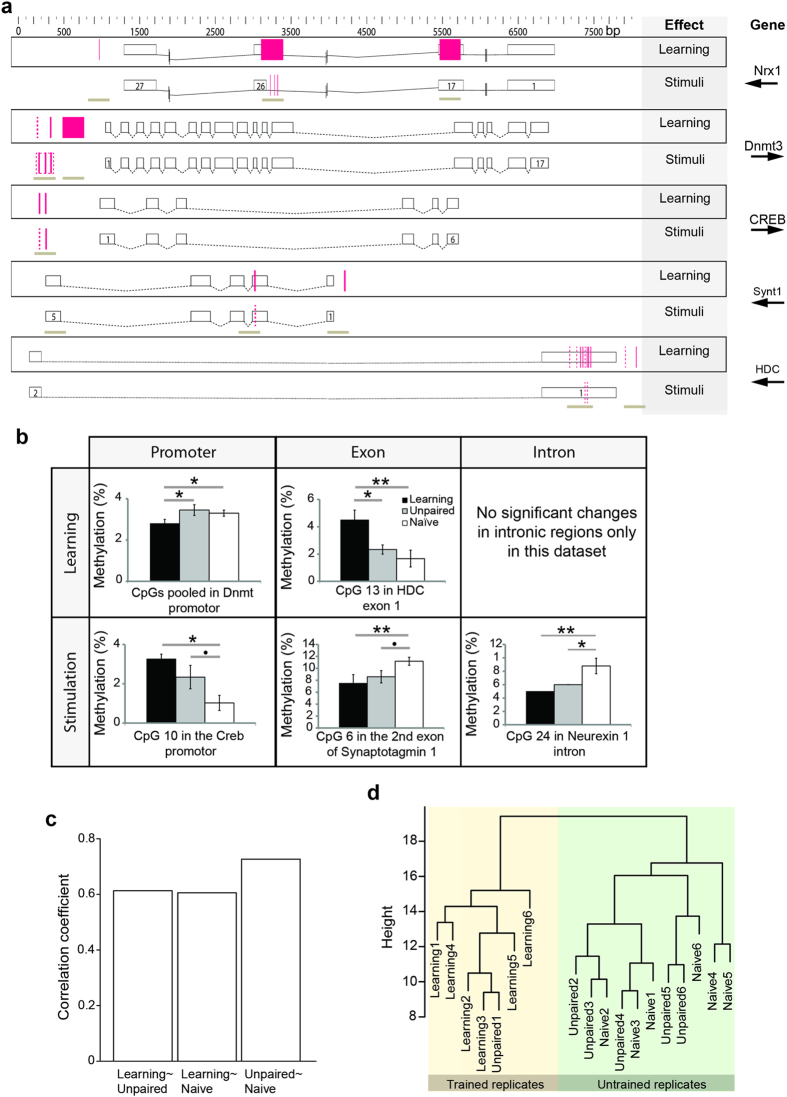
*Dnmts* and *Tet* show temporally distinct expression patterns during memory formation. Bees were trained and sacrificed 1, 3, 5 or 24 hours after training. Control bees were trained using an unpaired protocol. The relative expression rate (rER) of each gene was calculated by normalising to the housekeeping gene *RPL32* and the unpaired control. (**a**) The expression patterns of *Dnmt1b* and *Tet* were most similar and form a distinct cluster from *Dnmt3*, *Dnmt1a* and *Dnmt2* (agglomerative hierarchical clustering using Ward’s method and Euclidian distances as input). The heatmap shows the rER for each gene after normalisation. (**b**) *Dnmt1b* and *Tet* are both upregulated as early as 1 hour after training (Welch’s t-test p < 0.05), whereas *Dnmt3* is upregulated at 5 hours after training (Welch’s t-test p < 0.05). *Dnmt1a* and *Dnmt2* both do not show significant changes at any early time point. 24 hours after training *Dnmt1a*, *Dnmt1b* and *Tet* are downregulated (Welch’s t-test *Dnmt1b* p < 0.001, *Dnmt1a* and *Tet* p < 0.01*, Dnmt2* p < 0.1). (**c**) There was no common pattern of temporal expression in genes sensitive to DNMT inhibition (shown in Fig. 2b,c). *Actin* e.g. was upregulated 1 hour after training and downregulated 24 hours after, whereas *sesB* did not show a significant change in expression. Presented are the mean (+/− SEM). ( = p < 0.1; * = p < 0.05; ** = p < 0.01; *** = p < 0.001) N = 4 (1 hour after training both groups); N = 3 (3, 5 and 24 hours after training both groups).

We could show that DNA methylation changes after learning, that methylation is important for the formation of stimulus-specific long-term memory and that it influences expression of memory-associated genes. Interestingly *Dnmt3*, one of the three genes responsible for DNA methylation, was upregulated after Dnmt inhibition and DNA methylation levels changed in its promoter after learning. This could indicate a feedback loop in that *Dnmt3* itself is regulated by DNA methylation. In biology, the temporal control of feedback loops is crucial in order to avoid unstable trends towards extreme values. Therefore, we were interested in the time courses of *Dnmt* and *Tet* expression after classical conditioning, as those offer insights in the dynamics of the associated DNA methylation and demethylation pathways. We included all known *Dnmt*s and their counterpart, *Tet,* into the analysis, and measured their expression rates at four timepoints during memory consolidation from 1 to 24 hours after training (Fig. 4a,b). Honey bees possess four different *Dnmt* genes (*Dnmt1a*, *Dnmt1b*, *Dnmt2* and *Dnmt3*) and one *Tet* gene. Dnmt2 mediates tRNA methylation, while the other Dnmts mediate DNA methylation; thus Dnmt2 is not relevant for transcriptional control. In contrast, Tet mediates demethylation of methylcytosine. We found that *Dnmt1b* and *Tet* were upregulated 1 hour and downregulated 24 hours after training (Fig. 4a,b, Welch’s t-test p < 0.05). This is consistent with our finding that methylation was both increased and decreased after learning (Fig. 3b). Conversely, *Dnmt3* was upregulated 5 hours after training and went back to baseline after 24 hours (Fig. 4a,b, Welch’s t-test p < 0.05). These findings suggest that memory consolidation requires a temporally controlled sequence of DNA methylation and demethylation, involving different enzymes at different timepoints: Dnmt1b and Tet first, Dnmt3 later.

As shown in Fig. 2b, DNA methylation affected not only *Dnmt3* expression, but also that of several memory-associated genes. We studied expression changes over time (Fig. 4c) of those genes that were most affected by Dnmt inhibition (Fig. 2b,c; Cluster 1) and of *CREB*. We found that each gene had a characteristic expression pattern over time: *actin* expression was increased initially, and decreased back to baseline within 3–5 hours, *Eag* expression was decreased after 24 hours, *CREB* increased expression only late, at 5 hours, and returned to baseline after 24 hours and *Hdc*, *sesB* and *synaptotagmin1* did not show significant changes at the timepoints investigated here. Thus, there was no correlated temporal pattern of gene expression between *Dnmts* or *Tet* and putative DNA methylation sensitive genes. Rather our data suggest a more complex transcriptional pattern than a “simple” increase during the first hours and decrease 24 hours after training, as observed so far^12^,^13^,^14^,^35^. This pattern is likely mediated by an equally complex transcriptional regulation including a temporally orchestrated sequence of DNA methylation and demethylation processes, as shown here, which contribute to, but not solely determine the transcriptional pattern after learning.

## Discussion

A stable association between a conditioned stimulus (CS, e.g. an odour) and an unconditioned stimulus (US, e.g. a sugar reward) will reliably evoke an appropriate behavioural response (CR, e.g. proboscis extension response, PER) to the CS. But even such a “simple” classical conditioning experiment induces many changes in the brain, including different associative memory traces^8^, modifications in primary odour-processing^36^,^37^,^38^ and shifts in the generalization pattern^33^. Epigenetic mechanisms allow neurons to regulate and stabilize changes in the brain during long-term memory formation at a transcriptional level. In honey bees, methylation of cytosines in the DNA is necessary to stabilize olfactory generalization, i.e. to allow associative memories to be specific for a particular odour^29^. In this study, we confirm that DNA methyltransferases (Dnmts) mediate the stimulus-specificity of memory retention 24 hours after training (Fig. 1). Furthermore, we examine which genes are involved (Fig. 2), their temporal expression patterns (Fig. 4), and study which genomic regions are differentially methylated (Fig. 3). The data indicate that memory formation includes a temporally staggered activation of DNA methylation and demethylation pathways.

We investigated a total of 30 memory-associated genes, and found that 9 were significantly upregulated in response to Dnmt inhibition, whereas none were downregulated. The genes investigated here were found to be most strongly differentially expressed, and mostly down regulated, in an earlier study using a similar learning paradigm^14^. We therefore speculate that DNA methylation induced by odor-reward learning may lead to the downregulation of a subset of memory-associated genes. This assumption is in agreement with earlier observations of a negative correlation between DNA methylation and gene expression^22^. However, we do not know yet whether those Dnmt sensitive genes are directly regulated by DNA methylation or whether the effect is indirect (e.g. DNA methylation regulating transcription factors which target these genes). Further, genome wide, analyses of methylation patterns is needed to examine this question. Genes sensitive to Dnmt inhibition (e.g. *Hdc*, *actin*, *sesB*, *neurexinI*, *synaptotagmin1*) (Fig. 2b,c) are involved in memory formation and olfaction in invertebrates via different mechanisms: dendrite pruning (*Hdc*), dendritic spine formation and elimination (*actin*), energy metabolism during neurotransmission (*sesB*), cell adhesion and signaling at the presynaptic terminal (*neurexinI*) and neurotransmitter release (*synaptotagmin1*)^14^,^15^,^39^,^40^,^41^,^42^,^43^,^44^,^45^,^46^. The fact that learning induces long-term downregulation of these genes (as indicated by their increased expression when Dnmts are inhibited and by previous studies^12^,^13^,^14^) suggests that memories may be stabilized, or normalized, by reducing synaptic remodeling. As inhibition of Dnmts in our study led to an upregulation of synaptic genes, it is possible that DNA methylation contributes to the stabilization of memories by restricting the expression synaptic genes.

However, it is implausible that learning exclusively leads to a decrease in synaptic gene expression, as this would counteract synaptic plasticity. Therefore, we hypothesize that an early upregulation of synaptic genes (e.g. *actin* in Fig. 4c) is followed by methylation-mediated regulation, which normalizes expression levels again. Indeed, experience induced normalization was observed at the level of olfactory receptors on the bees’ antennae^34^. Neuronal and synaptic normalization mechanisms are necessary because otherwise positive feedback loops could become rampant when constantly processing new environmental information. Our observation that methylation is necessary to reduce olfactory generalization confirms its role as a normalization mechanism, as does the observation that methylation is necessary for memory extinction in honey bees^28^. Normalization mechanisms are crucial for proper brain function not only in insects. Impaired normalization results in over-connectivity and over-activity of neurons in mammals, which in turn causes memory impairment and reduced discriminatory ability in schizophrenia, autism disorders and ageing^47^,^48^,^49^,^50^.

Arguably the similarity of the *Dnmt* gene family between mammals and bees is surprising, given that *Dnmt3* and *Dnmt1* are absent in other organisms such as *Drosophila melanogaster*^51^. Despite the genetic relatedness between the honey bee and mammalian DNA methylation machinery, in this study we show important functional differences. We show that in bees, *Dnmt1b* is upregulated after training (Fig. 4a,b), while in mice Dnmt1 is not involved in learning or in aging-related cognitive decline^52^,^53^. This suggests that either Dnmt1 is important in bees, but not mammals, during memory formation or indeed Dnmt1 and Dnmt3 have different functional roles in DNA methylation across species than previously suggested^54^. It will be important to analyse honey bee Dnmt1a/b and Dnmt3 concerning their methylation preferences for either hemi- or unmethylated cytosine to further understand their individual role during learning.

Memory formation induces a complex and long-lasting pattern of gene regulation. Our results show that there is a temporal pattern in *Dnmt* and *Tet* expression: classical conditioning of an odour leads to *Dnmt1b* and *Tet* being upregulated first (after 1 hour), and *Dnmt3* later (after 5 hours, Fig. 4a,b). This temporal complexity corroborates our assumption that learning induced changes may be followed by memory consolidation which consists, in part at least, in normalization processes potentially involving DNA methylation-dependent gene regulation. Consequently, we are not surprised to see that the temporal pattern of upregulated and downregulated genes depends on the learning paradigm used: in differential training, *Dnmt3* is already upregulated 30 minutes after training^28^.

Although the expression of the DNA methylation and demethylation machinery is most dynamic during the first hours after training and is at baseline or below after 24 hours, DNA methylation changes found here were stable (Fig. 3a,b). Stable changes in DNA methylation patterns have been shown to occur in specific genomic regions of rats after training as well^55^. This suggest that learning impacts the DNA methylation pattern permanently and therefore may also have a permanent effect on gene expression. Alternatively it has been suggested previously that stable DNA methylation changes and epigenetic modifications in general, could serve as tags for a rapid reactivation of previously active genes, rather than having a permanent effect on gene expression^56^,^57^. This theory is especially compelling as it explains how the need for plastic responses to the environment is accommodated within stably functioning neurons, at a transcriptional level. Besides gene expression, alternative splicing has also been associated with DNA methylation^20^,^22^,^24^,^58^,^59^. It will be an important aim for future research to unravel the relationships between DNA methylation, gene expression and alternative splicing in behavioural plasticity.

In order to gain additional information about which neuropils are involved in stimulus-specific long-term memory, it would be necessary to perform the measurements reported here separately for different brain areas (e.g. antennal lobes, mushroom bodies, lateral protocerebrum). However, some speculation is possible: in a recent model of the olfactory system, the lateral protocerebrum was proposed as the site for associative value memory (i.e. the ‘meaning’ of an odor e.g. food vs. no food), while the mushroom bodies were proposed for associative odour identity learning^60^. Here, we could dissociate the associative odour component (associative value) and the odour identity component (generalization) by interfering with methylation. We hypothesize that the cellular mechanisms associated with methylation dependent memory are localized either directly in the mushroom bodies (odour identity) or in neurons conveying information to the mushroom bodies. Indeed, some genes affected here have already been shown to have high expression rates in the mushroom bodies^15^,^16^.

Just as DNA methylation, its counterpart demethylation is equally important for long-term memory (Fig. 3a,b). Clearly, there is a dynamic relationship between different methylation pathways in the brain. Therefore a balance between opposing methylation processes is likely required to create an appropriate neuronal response. However, the enzyme investigated here, Tet, may not be the only player involved: there are additional enzymes which can act as demethylases (e.g. Gadd45a)^61^ and under specific experimental conditions also Dnmts can have demethylation activity^62^. Another aspect of DNA methylation is which sites are targeted. Our analysis was restricted to CpG target sites. However, non-CpG methylation and hydroxymethylation are additional cytosine modifications in bees^19^,^20^. Therefore future studies will need to analyse the global methylation and hydroxymethylation patterns to gain a genomic perspective concerning the type and number of targets sites during memory formation. Other epigenetic mechanisms also play a role in memory formation in honey bees, including microRNAs and histone modifications^14^,^63^. Different epigenetic mechanisms likely interact: the structural gene *actin* is a key molecule for neuroplasticity, and is regulated by miR-932 after training^14^, besides being sensitive to Dnmts (this study). Thus, DNA methylation, histone modifications and non-coding RNAs interact and influence each other^64^. It will be a welcome challenge for future research to untangle the specific contributions that each of these epigenetic mechanisms has on memory formation when considering the significant task of identifying respective targets and elucidating the temporal scales of their activity.

In summary, we show that long-term memory formation in honey bees induces a temporally complex pattern of demethylation and methylation of genes over the first 24 hours after training. Many of these genes regulate neural networks via dendrite formation, synapse morphology, energy metabolism and neuron excitability. Importantly, we also show that genetic control involves feed-back regulation: genes encoding the DNA methylation and demethylation machinery (*Dnmt1b*, *Dnmt3* and *Tet*) are themselves up- and downregulated after training, in a sequential manner. Additionally, the *Dnmt3* promoter was differentially methylated in response to learning. These results clarify some aspects on how epigenetic gene regulation contributes to a long-term engram, while adding important new questions to our search for how brains can encode persistent memories.

## Methods

Three main experiments were performed in this study. For each experiment bees were trained using classical appetitive olfactory conditioning.

### Olfactory conditioning and Brain dissections

Experiments were performed using one outdoor hive at the Queensland Brain Institute, University of Queensland, Australia. A brood frame was removed from the hive and kept in a humid incubator at 37 °C. Every 24 hours newly hatched bees were marked with enamel paint (Tamiya, Japan) on their thorax and returned to a smaller hive, originating from the original experimental hive. After 10 days bees were collected, mounted into plastic tubes and fixed, so that the thorax was still accessible. They were fed with 1 M sugar water and kept overnight in an incubator at 27 °C. The next day bees were trained with 6 trials of 1-hexanol (Sigma-Aldrich, St. Louis, USA; 1:100 in hexane) as conditioned stimulus (CS). The CS was presented for 4 s and 3 s of 1 M sugar reward (US) were given. Odour and sugar stimuli were overlapping by 2 s. The intertrial interval was 10 minutes. Bees were kept in the incubator and fed with 1 M sugar water, if kept overnight. In every experiment all groups were trained in parallel.

### Dnmt inhibition experiment

Bees were treated 2 hours after training with either 1 μl of the Dnmt inhibitor Zebularine, RG108 (both 2 mM in DMF, Sigma-Aldrich, St. Louis, USA) or the solvent DMF (Sigma-Aldrich, St. Louis, USA) topically on the thorax, as described previously^28^,^29^, or were left untreated. 24 hours after training bees were tested for CS memory retention and response to a novel odour (Sigma-Aldrich, St. Louis, USA; 1-nonanol, 1:100 in hexane). The order of CS and novel odour in the test was balanced. After the test brains were immediately dissected by freezing bees and dissecting the central part of the brain (all except the optical lobes). All bees responding to sugar water during the training and after the test were analysed. All bees within one behavioural replicate matching these criteria were pooled (3–8 brains per replicate). Replicates with less than 3 bees matching the criteria were discarded.

### Timeline experiment

Brains were dissected 1, 3, 5 or 24 hours after the training as above. Bees were either trained as described above or an unpaired paradigm was used (CS and US separated by 5 minutes). Additionally to sugar responsiveness only bees learning the odour during training (PER > 2 ×, learner group), respectively not learning (PER < 3 ×, unpaired group) were used, as bees were not tested for memory retrieval.

### Methylation events experiment

Bees were trained as described above. As control an unpaired paradigm was used and an air only ‘naïve’ control. Brains were dissected after the test as described above.

### Gene expression

RNA was extracted using Trizol (Ambion, Kassel, Germany) as recommended by the manufacturer. Concentration of RNA (as well as DNA and biDNA) was measured with the Nanodrop (Thermo Fisher Scientific, Waltham, USA). Gene expression was analysed using the Fluidigm system (Fluidigm, San Francisco, USA). In short cDNA was synthesized from 2 μg RNA using Superscript III (Invitrogen, Carlsbad, USA). After cDNA synthesis, cDNA was pre-amplified with a mix of all primers (Full list: Table S1) using Taqman Pre-amplification Mix (Taqman, Carlsbad, USA) according to the Fluidigm manual. Fluidigm runs included 43 or 47 primer assays and were analysed with a 48 × 48 GE Chip (Fluidigm, San Francisco, USA). Samples belonging to the same experiment were run on the same Chip always. If one sample was an outlier or not detectable in 30% or more of all primer assays it was excluded from the analysis.

### Methylation analysis

DNA was extracted using Trizol as recommended by the manufacturer. DNA was bisulfite treated using the EZ bisulfite kit (Zymo, Irvine, USA) as recommended by the manufacturer with few changes: samples were incubated with CT conversion reagent for 18 cycles with 30 s at 95 °C and 15 min at 50 °C each time; bisulfite treated DNA (biDNA) was washed 5 times with 10 μl H_2_O. Primer for the Sequenome analysis were designed using Epidesigner (Sequenome Inc., San Diego, USA) (Primerlist: Table S2; Amplicon predictions: Figure S1) based on the beebase Amel_4.0 genome assembly. We followed the Sequenome workflow as recommended by the company. In brief, biDNA was amplified using optimized conditions for each primer. After cleavage and cleaning, samples were dispensed onto a SpektroChip with a Nanodispenser (both, Sequenome Inc., San Diego, USA). The Chip was then run on the Sequenome platform using default settings. Data was checked for outliers using boxplots and outliers were removed before analysis. The bisulfite conversion efficiency was analysed for every sample using the MassArray R-pipeline^65^ build-in conversion control script (Figure S2).

### Global DNA methylation

DNA was extracted as described above and diluted to 100 ng/μl. 1 μl of DNA was used for measuring global DNA methylation. Global DNA methylation was assessed using the Methylamp Global DNA methylation Colorimetric Quantification Kit (Epigentek, Farmingdale, US) according to the companies’ recommendations.

### Statistical analysis

Behavioural experiments were analysed using a McNemar test to compare the response to the CS + and new odour within one treatment group, as each bee was presented with both odours. The McNemar test is appropriate for paired binary data. The discrimination indexes were calculated by subtracting the test response to the CS from the test response to the new odour for every individual. The data was tested using a generalized linear model with factor treatment. Gene expression experiments were analysed in the following way: Data was normalized using the method described by Schefe *et al.*^66^ resulting in a relative expression rate (rER). *RPL32* was used as housekeeping gene in all experiments. Log-transformed and normalized values were used for statistical analysis. Data was either analysed with a generalized linear model (glm) if two or more groups were compared and otherwise with a one-sided t-test. Global DNA methylation was analysed accordingly. Methylation sites were analysed both pooled and individually. For the pooled analysis all CpGs were pooled per replicate and group. First a dissimilarity matrix was calculated (R-package ‘daisy’ with setting ‘Euclidian’). The dissimilarity matrix was then used to calculate clusters using an agglomerative hierarchical clustering approach (R-package ‘agnes’ method ‘ward’). Second, pooled CpGs were correlated between the three groups (Learner, Unpaired and Naïve) (R-package ‘corr’ method ‘spearman’). Individual amplicons were tested using generalized linear models as described above with Group (Learner, Unpaired, Naïve), Age (11, 13 days) and Season (autumn, spring) as factors. Anova was used to test the glm model for effective factors.

## Additional Information

**How to cite this article**: Biergans, S. D. *et al.* *Dnmts* and *Tet* target memory-associated genes after appetitive olfactory training in honey bees. *Sci. Rep.* **5**, 16223; doi: 10.1038/srep16223 (2015).

## Supplementary Material

Supplementary Information
