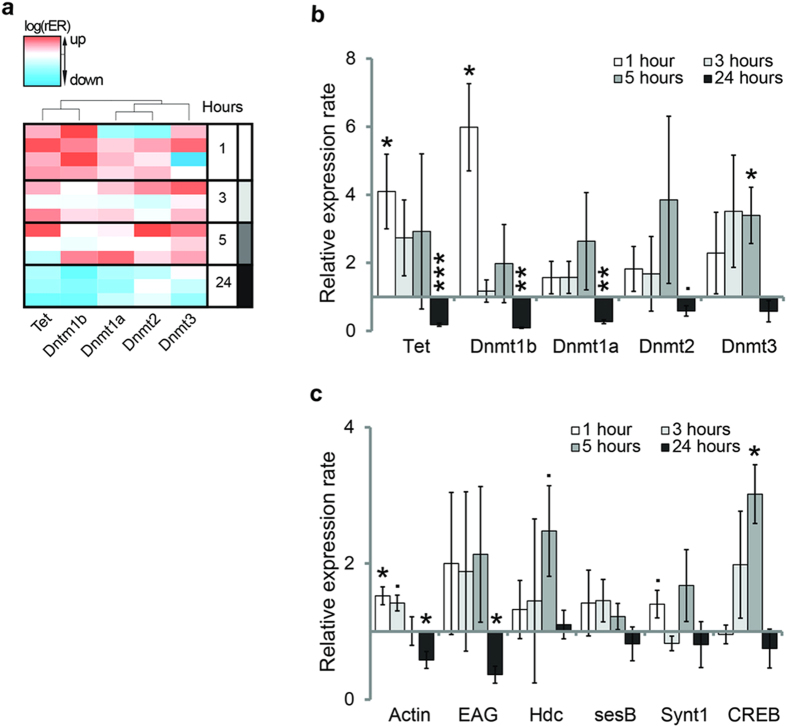# Corrigendum: *Dnmts* and *Tet* target memory-associated genes after appetitive olfactory training in honey bees

**DOI:** 10.1038/srep21656

**Published:** 2016-02-25

**Authors:** Stephanie D. Biergans, C. Giovanni Galizia, Judith Reinhard, Charles Claudianos

Scientific Reports 5: Article number: 1622310.1038/srep16223; published online: 11042015; updated: 02252016.

This Article contains an error in the order of the Figures. Figures 3 and 4 were published as Figures 4 and 3 respectively. The correct Figures 3 and 4 appear below as Figs 1 and 2 respectively. The Figure legends are correct.

## Figures and Tables

**Figure 1:**
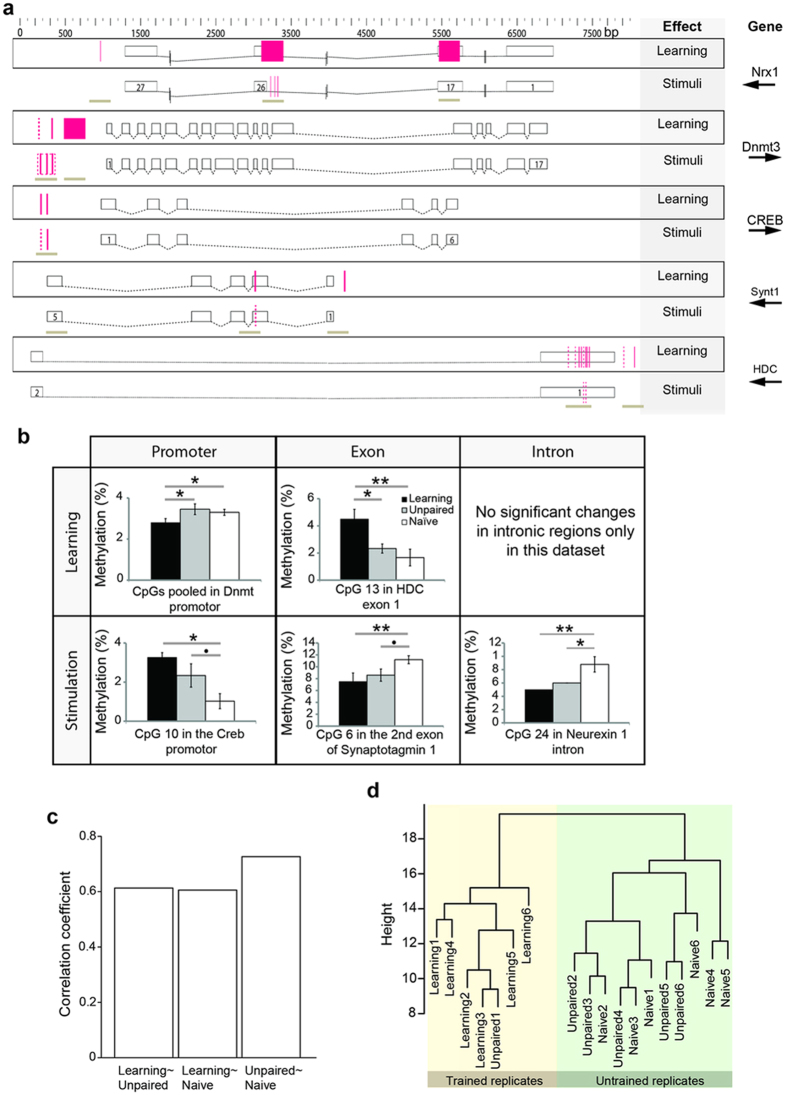


**Figure 2:**