# Supplementary material for: Dnmts and Tet target memory-associated genes after appetitive olfactory training in honey bees
Source: Sci Rep. 2015 Nov 4;5:16223. doi: 10.1038/srep16223 (PMC4632027; doi:10.1038/srep16223)
Supplement: Supplementary Information [file srep16223-s1.pdf]

# *Dnmts* and *Tet* target memory-associated genes after appetitive olfactory training in honey bees

Stephanie D. Biergans[1,2*], C. Giovanni Galizia[2], Judith Reinhard[1], Charles Claudianos[1,3]

[1] Queensland Brain Institute, The University of Queensland, Australia

[2] Neurobiologie, Universität Konstanz, Germany

[3] Monash Institute of Cognitive and Clinical Neuroscience, Faculty of Biomedical and Psychological Sciences, Monash University, Australia

*Corresponding author: stephanie.biergans@uq.net.au

## SUPPLEMENTAL MATERIALS

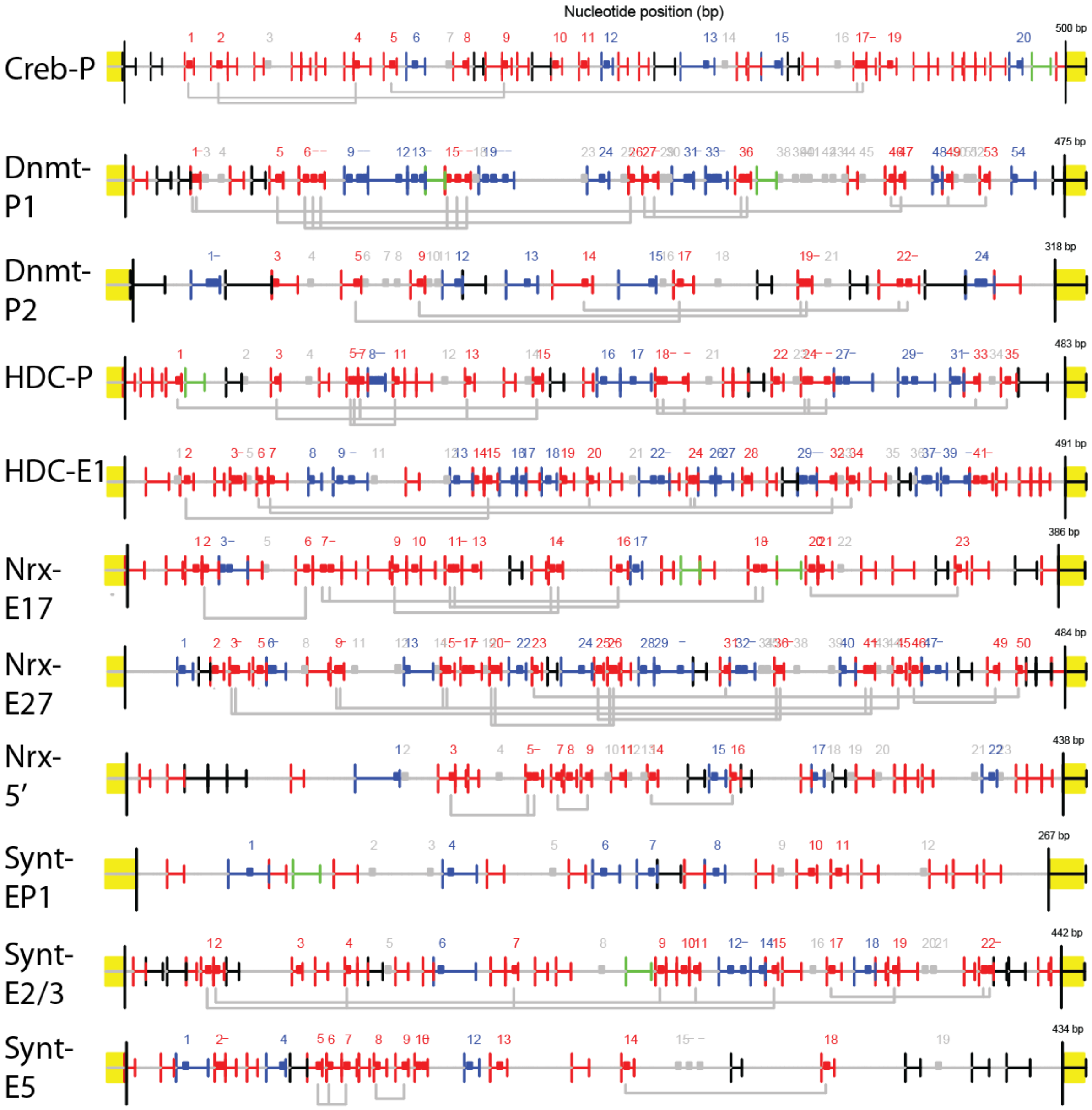


Figure S1 Amplicon prediction for Sequenome analysis. The amplicon prediction function of the MassArray R-pipeline was used (Thompson et al., 2009). Numbers represent CpG sites. Red indicates CpGs which cannot be analysed, because the respective fragment mass overlaps with another fragment. Grey CpGs are on fragments which are out of the measurable range. Blue CpGs can be analysed. Green parts are segments which were used for the conversion control analysis as they include non-CpG cytosines.

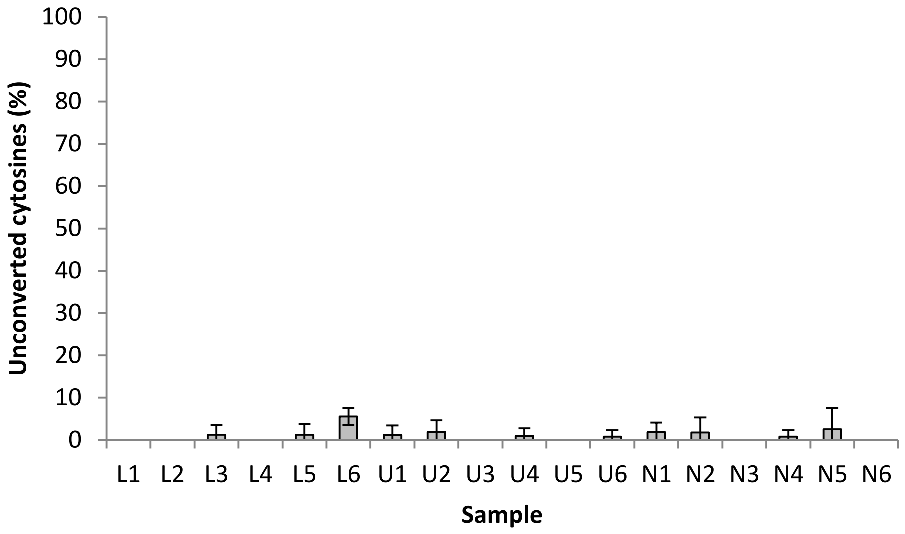


Figure S2 Bisulfite conversion control for Sequenome analysis. The conversion control pipeline of the MassArray R-pipeline was used (Thompson et al., 2009). For all amplicons conversion of non-CpG C's was assessed and pooled per sample. Mean and 95% confidence interval are presented. L= learner group; U= unpaired group; N = naïve group

Table S1 Fluidigm qPCR primer list

| Gene candidate | GB number (Assembly 4.0) | Forward primer | Reverse primer |
|---|---|---|---|
| Actin | 17681 | TTCCCATCTATCGTCGGAAG | CTCTCTTTGATTGGGCTTCG |
| Aldoreductase | 18109 | TAGTCCCCTTGGATCACCTG | TTGGGTCATCTGGTTTAGCC |
| Asparaginesynthetase (ASNS) | 13219 | TGGAATTTGGGCTCTTTTTG | TTCTGGACCACGGTGTGTAA |
| Histone acetyltransferase p300 (HAT p300) | 10171 | ACCAAGTGGAGGTCAACCTG | ATATTGTGGGTGGGCAAGAA |
| cAMP response element binding protein (CREB) | 11585 | AATTGCAACCCAAGGTGAAG | TCAGTATGCACAAGGCCAAG |
| Cueball (cue) | 17323 | CCAAAAGACGGGAAAAATGA | ACGCGTTAAAATCCCACTTG |
| DNA methyltransferase 1a (DNMT1a) | 19865 | TGATCCAAAAACAGATGAGGA A | TACAGCACCATTCGGATGAC |
| DNA methyltransferase 1b (DNMT1b) | 15130 | GAAATTACATGGGTGGGAGAA | GTCACTGCCTCTTCGAAACC |
| DNA methyltransferase 2 (DNMT2) | 10767 | TGAGTCCTCCATGTCAACCTT | GCCAAATTGACAAGGGCTTA |
| DNA methyltransferase 3 (DNMT3) | 14232 | CCTCCAACTGGACTTTGGAC | ACGTTCGGATTGTCCTTCAG |
| Ether a go-go (EAG) | 15597 | GATGACCAAGGGCCTAGACA | ATGCTCGTTGAACACCTTCC |
| Glyceraldehyde-3-phosphate | 14798 | GATGCACCCATGTTTGTTTG | TTTGCAGAAGGTGCATCAAC |

| | | | |
|---|---|---|---|
| dehydrogenase (GAPDH) | | | |
| GB18684 | 18684 | TATTGTTGCTTCGGATCGTG | CGGTGTTCTTTTTCCGATCT |
| Histone 1B | 12700 | GCTAAGAAGCCAGCAACACC | TACCTTCGATGCGCTCTTTT |
| Headcase (HDC) | 11140 | ATCCGGGAAGAGGAAGTGAT | TGTTCTCCATGGTGTCGTGT |
| Histone 1.2 | 12218 | GCCAATCCAACAAAGAAAGC | ATTGGTGACCGTCGTGATTT |
| Heatshockprotein 90 (HSP90) | 14758 | GGCTGCCAAGAAGCATTTAG | AGCTTCAGCTTTTTGCCTCA |
| cytoplasmic aconitate hydratase (Irp1) | 16072 | TATCGGAAAAGCTGGACCAC | TATCGGAAAAGCTGGACCAC |
| Molybdenum cofactor sulfurase (mal) | 18250 | CATCCTTGCTAATGCTTCACC | GCAACAATTCCTCCTTGCAT |
| molybdenum cofactor biosynthesis protein 1 (Mocs1) | 11214 | TTGTATGCCAGCAGAAGGAA | TTGGTTCACCACCAGTTAAGC |
| Mitochondrial ribosomal protein L35 (Mrpl35) | 14476 | AACTTTTGCTGGCCGTCAT | AACTTTTGCTGGCCGTCAT |
| Neprilysin 2(Nep2) | 16619 | TTGAAGAACAGCACGAAACG | ATAATCCAAGAGCGGCTTGA |
| Neurexin I | 13455 | CGGAGAACGCTGCCTTAATA | TCAGAACAATGGCGATCAAC |
| N-acetylneuraminate lyase(NPL) | 17289 | TAGTGAGGCAGCTCCAAACA | CGAGGAATTCTCCCATGTGT |
| Pyridoxamine 5'-phosphate oxidase (PPO) | 13619 | TGTGGAGGCAGAGCCTTAGT | TACACCGTCGCAAAAACTGA |
| RNA-polymerase (Rpb8) | 10191 | TATCCTGATGGAGGGGAGTG | GGGTTCATTGCTTGCTTCAT |
| Ribosomal protein L32 (RPL32) | 10903 | CGTAACCTTGCACTGGCATT | TTGCTCATGGTGTGAGCAGT |
| Regulatory particle non-ATPase 9 (RPN9) | 16614 | CAAGCTTTCAATGCTGGTGA | CTTTGCTCCATTGTGGCTTT |
| Sec61beta | 13924 | CCCAGCAAAGCTATAGCACCT | TTCATCAGAACCAGCTCCTGT |
| Stress sensitive B (sesB) | 17499 | TGATGTAGGCAAAGCTGGTG | GGCAGCACGATAGATGATGA |
| Synaptotagmin 1 (Synt) | 20036 | CCAAACACGATCAGATCGGTG | CTTCAGATCCGTACGTGAAGG |
| Ten-eleven translocation methylcytosine dioxygenase (Tet) | 13880 | TCACGAGCAAAAGACACCTGG | ACATGTTTTCCGGCTTATCG |

Table S2 Sequenome primer list

| Primer name | Forward | Reverse | Size (bp) |
|---|---|---|---|
| CREB Promotor | aggaagagagTTGGTAAGTATTTAGTTGGAGAATAA | cagtaatacgactcactatagggagaaggctAACATCATCAAAACAAATAATATC | 500 |
| Dnmt3 Promotor 1 | aggaagagagGAAATTTGGGAAGAAAGTGGG | cagtaatacgactcactatagggagaaggctAAAAATTCTCTTCATCAAAAAAAAC | 475 |
| Dnmt3 Promotor 2 | aggaagagagGTTTTTTTTGATGAAGAGAATTTTT | cagtaatacgactcactatagggagaaggctAAATTCTTACCTAACCACCATCCC | 318 |

| | | | |
|---|---|---|---|
| HDC Promotor | aggaagagagGTTTAAAATTGTTATTTGAAAGTGT | cagtaatacgactcactatagggagaaggctCTATATTATCACACACACACCCATT | 483 |
| HDC Exon 1 | aggaagagagGAAAAGTATAAGGGTGTGTTTGATA | cagtaatacgactcactatagggagaaggctTTCCTAATAACATCCAATCCAAATC | 491 |
| Neurexin 1 exon/intron 17 | aggaagagagTTTGGTAAATAGAGTATTTTGGAATTT | cagtaatacgactcactatagggagaaggctAAAACATATCCAAACAATTTTCCCTAT | 386 |
| Neurexin 1 exon/intron 26 | aggaagagagGAAATTGAAAAAGGAAAGTAATTAATAG | cagtaatacgactcactatagggagaaggctAAAAACTAACAAACTCACCTAAC | 484 |
| Neurexin 1 5'end | aggaagagagGAGAAATTTGATTGAGGAATGTGTT | cagtaatacgactcactatagggagaaggctTTCCTAACCTCAAACCTCAAATATC | 438 |
| Synaptotagmin 1 Promotor/exon 1 | aggaagagagATTTAAAGATGGGAAGAAAGGATTG | cagtaatacgactcactatagggagaaggctTTTTACCTTATATTAAAATTTCCCA | 267 |
| Synaptotagmin 1 exon 2/3 | aggaagagagGGAATTGTTAGTTTTGGATATGGGT | cagtaatacgactcactatagggagaaggctCCAATTCAAATTTACCTAACCACCT | 442 |
| Synaptotagmin 1 exon 5 | aggaagagagGGTATATTAGAATTTATTGGGAAGG | cagtaatacgactcactatagggagaaggctATTCTTTTCTCTCATAAAAAAAACCTA | 434 |

Table S3 p-values methylation site analysis

**LEARNING** **STIMULATION**

| Amplicon | CpG | L~U | L~N | U~N | Group | Age | Season |
|---|---|---|---|---|---|---|---|
| Creb Promotor | All | 0.969 | 0.740 | 0.675 | 0.883 | 0.436 | 0.082 |
| | 6 | 0.432 | 0.136 | 0.397 | 0.111 | **0.010** | 0.696 |
| | 8 | 0.754 | 0.425 | 0.593 | 0.733 | 0.423 | 0.800 |
| | 10 | 0.244 | **0.018** | ***0.086*** | **0.006** | 0.754 | 0.059 |
| | 11 | 0.919 | 0.338 | 0.352 | 0.535 | 0.320 | 0.612 |
| | 12 | 0.337 | 0.951 | 0.332 | 0.525 | 0.463 | 0.429 |
| | 13 | 0.587 | **0.017** | **0.023** | **<0.001** | ***0.072*** | 0.920 |
| | 15 | 0.348 | 0.820 | 0.22 | 0.126 | 0.101 | **0.004** |
| | 19 | 0.863 | 0.311 | 0.358 | 0.241 | **0.014** | ***0.058*** |
| | 20 | 0.321 | 0.503 | 0.735 | 0.628 | 0.629 | 0.587 |
| Dnmt 3 Promotor 1 | All | 0.106 | 0.608 | ***0.054*** | 0.115 | 0.705 | 0.982 |
| | 9.10.11 | 0.744 | 0.828 | 0.608 | 0.882 | 0.530 | 0.526 |
| | 12 | ***0.071*** | 0.396 | 0.309 | 0.184 | 0.659 | 0.708 |
| | 13.14 | 0.265 | 0.207 | **0.037** | **0.015** | **0.046** | 0.102 |
| | 19.20.21.22 | 0.252 | 0.614 | 0.131 | 0.193 | 0.203 | 0.178 |
| | 24 | 0.222 | 0.298 | **0.046** | 0.119 | 0.762 | 0.782 |
| | 31.32 | 0.757 | **0.042** | **0.031** | **0.031** | 0.328 | 0.867 |
| | 33.34.35 | 0.692 | 0.321 | 0.559 | 0.569 | 0.253 | 0.323 |
| | 54 | 0.143 | 0.143 | 1 | 0.193 | 0.364 | 0.454 |
| Dnmt 3 Promotor 2 | All | **0.045** | ***0.093*** | 0.606 | ***0.077*** | 0.524 | 0.694 |
| | 3 | 0.249 | 0.731 | 0.380 | 0.477 | 0.707 | 0.225 |
| | 11.12 | ***0.092*** | 0.618 | 0.161 | 0.1850 | 0.457 | 0.787 |

| | | | | | | | |
|---|---|---|---|---|---|---|---|
| | 13 | 0.259 | 0.455 | 0.763 | 0.465 | 0.696 | 0.188 |
| | 15 | ***0.086*** | **0.006** | 0.163 | **0.002** | 0.387 | 0.281 |
| | 24.25 | **0.022** | **0.046** | 0.6059 | **0.003** | 0.283 | **0.019** |
| HDC Promotor | All | 0.841 | 0.346 | 0.454 | 0.595 | 0.340 | 0.343 |
| | 8.9.10 | 1 | 1 | 1 | 1 | 0.464 | 0.534 |
| | 16 | **0.031** | 0.109 | 0.506 | ***0.071*** | 0.696 | 1 |
| | 17 | 0.835 | 0.533 | 0.677 | 0.828 | 0.511 | 0.908 |
| | 22 | 0.831 | 0.114 | 0.164 | 0.213 | 0.807 | 0.359 |
| | 27.28 | 0.169 | 0.770 | 0.113 | 0.165 | 0.821 | **0.048** |
| | 29.30 | 0.121 | ***0.093*** | 0.946 | 0.169 | 0.805 | 0.654 |
| | 31.32 | 0.868 | 0.679 | 0.804 | 0.918 | 0.298 | 0.704 |
| HDC exon 1 | All | 0.732 | 0.827 | 0.901 | 0.932 | **0.046** | 0.921 |
| | 3.4 | 0.692 | 0.432 | 0.692 | 0.554 | 1 | **<0.001** |
| | 8 | 1 | 1 | 1 | 1 | 1 | 1 |
| | 9.10 | 0.897 | 0.146 | 0.132 | 0.226 | 0.333 | 0.629 |
| | 13 | **0.018** | **0.004** | 0.428 | **0.002** | 0.420 | 0.494 |
| | 14 | 0.143 | 0.292 | 0.772 | 0.328 | 0.926 | 0.957 |
| | 16 | 0.502 | **0.029** | 0.106 | ***0.060*** | 0.720 | 0.448 |
| | 18 | **0.003** | ***0.195*** | ***0.051*** | **0.003** | 0.831 | 0.711 |
| | 19 | ***0.079*** | 0.935 | ***0.079*** | ***0.100*** | 0.240 | 0.683 |
| | 22.23 | **0.024** | 0.342 | 0.119 | **0.049** | 0.315 | 0.845 |
| | 26 | **<0.001** | **<0.001** | 0.218 | **<0.001** | 0.146 | 0.769 |
| | 27 | 0.503 | 0.503 | 1 | 0.715 | 0.885 | ***0.082*** |
| | 28 | 0.800 | 0.529 | 0.384 | 0.499 | **0.009** | 0.720 |
| | 29.30 | ***0.064*** | ***0.088*** | 0.797 | ***0.099*** | 0.882 | 0.376 |
| | 37.38 | 0.189 | 0.189 | 1 | 0.221 | **0.041** | 0.385 |
| | 39.40 | 0.833 | 0.521 | 0.686 | 0.794 | 0.117 | 0.831 |
| | 41.42 | 0.141 | ***0.073*** | 0.694 | 0.151 | 0.501 | 0.528 |
| Neurexin 1 exon/ intron 17 | All | **0.038** | 0.104 | 0.687 | **0.004** | 0.484 | **<0.001** |
| | 1 | 0.399 | 0.343 | 0.870 | 0.610 | 0.794 | 0.523 |
| | 3.4 | 0.431 | 0.164 | 0.431 | 0.401 | 0.898 | 1 |
| | 10 | 0.124 | 0.112 | 0.734 | ***0.053*** | 0.120 | **0.031** |
| | 13 | 0.241 | 1 | 0.241 | 0.325 | 0.920 | 0.131 |
| | 21 | **0.050** | ***0.098*** | 0.826 | **0.002** | 0.654 | **<0.001** |
| Neurexin 1 exon/ intron 26 | All | 0.246 | **0.017** | 0.124 | **0.014** | 0.584 | ***0.060*** |
| | 1 | 0.457 | 0.379 | 0.121 | 0.305 | 0.636 | 0.969 |
| | 6.7 | 0.125 | 1 | 0.147 | **0.016** | 1 | **<0.001** |
| | 12 | 0.151 | 0.114 | 0.810 | 0.176 | 0.682 | 0.158 |
| | 13 | 0.721 | **0.039** | **0.050** | **0.031** | 0.419 | 0.209 |
| | 17.18 | 0.558 | 0.126 | **0.034** | ***0.060*** | 0.681 | 0.225 |
| | 22 | 0.718 | 0.441 | 0.652 | 0.634 | 0.542 | **0.007** |
| | 24 | 0.467 | **0.015** | **0.039** | **0.006** | 0.888 | 0.204 |
| | 28 | 0.823 | 0.301 | 0.379 | 0.554 | 0.502 | 0.609 |
| | 29.30 | 0.120 | 0.503 | 0.323 | 0.169 | 0.443 | ***0.088*** |
| | 32.33 | 0.115 | 0.510 | 0.328 | 0.137 | **0.048** | 0.121 |
| | 40 | 0.307 | 0.070 | 0.268 | 0.175 | 0.560 | 0.956 |
| | 47.48 | 0.971 | 0.773 | 0.708 | 0.927 | 0.838 | 0.346 |
| Neurexin 1 5’ end | All | 0.194 | 0.160 | 0.845 | 0.199 | 0.182 | 0.219 |
| | 1 | 0.340 | 0.267 | 0.827 | 0.282 | **0.007** | 0.306 |
| | 8 | 0.594 | 0.155 | 0.352 | 0.289 | 0.254 | 0.215 |
| | 11 | **0.027** | 0.124 | 0.508 | **0.040** | 0.471 | 0.339 |
| | 15 | 0.328 | 0.709 | 0.591 | 0.601 | 0.693 | 0.213 |
| | 17 | 0.171 | 0.516 | 0.484 | 0.213 | **0.011** | 0.531 |
| | 22 | 0.370 | 0.535 | 0.751 | 0.639 | 0.331 | 0.703 |

| | | | | | | | |
|---|---|---|---|---|---|---|---|
| Synaptotagmin 1 exon/ intron 2 and 3 | All | 0.141 | 0.517 | 0.388 | 0.270 | ***0.092*** | 0.574 |
| | 3 | 0.391 | 0.280 | 0.061 | 0.155 | 0.740 | 0.612 |
| | 6 | 0.479 | **<u>0.031</u>** | ***0.093*** | **<u>0.004</u>** | **<u>0.028</u>** | **<u>0.030</u>** |
| | 10 | 0.131 | 0.131 | 1 | 0.235 | 0.612 | 0.570 |
| | 12.13 | 0.233 | 0.170 | 0.774 | 0.374 | 0.947 | 0.582 |
| | 14 | 0.292 | 0.547 | 0.676 | 0.513 | 0.114 | 0.332 |
| | 18 | 0.232 | 0.283 | 0.924 | 0.278 | ***0.051*** | ***0.079*** |
| Synaptotagmin 1 Exon/ intron 5 | All | 0.690 | 0.705 | 0.998 | 0.903 | 0.261 | 0.815 |
| | 1 | 0.718 | 0.404 | 0.250 | 0.507 | 0.577 | 0.387 |
| | 2.3 | 0.116 | 0.320 | 0.584 | 0.299 | 0.972 | 0.777 |
| | 4 | 0.401 | 0.174 | 0.554 | 0.387 | 0.457 | 0.738 |
| | 10.11 | 0.134 | 0.766 | 0.357 | 0.292 | 0.519 | 0.964 |
| | 12 | 0.923 | 0.658 | 0.555 | 0.812 | 0.234 | 0.293 |
| | 13 | 1 | 0.111 | 0.181 | **<u>0.045</u>** | 0.686 | **<u>0.017</u>** |
| Synaptotagmin 1 Promotor and exon 1 | All | 0.687 | 0.345 | 0.167 | 0.348 | 0.260 | 0.887 |
| | 1 | **<u>0.023</u>** | 0.112 | 0.389 | **<u>0.032</u>** | 0.444 | 0.165 |
| | 4 | 0.204 | 0.103 | 0.630 | 0.184 | 0.132 | 0.921 |
| | 6 | 0.689 | 0.689 | 1 | 0.895 | 1 | 0.104 |
| | 7 | 0.295 | 0.126 | 0.594 | 0.305 | 0.737 | 0.796 |
| | 8 | 0.177 | 1 | 0.152 | 0.172 | 0.123 | 0.253 |
| | 10 | 0.640 | 0.369 | 0.162 | 0.334 | 0.246 | 0.563 |
